# Effect of Selected Thiols on Cross-Linking of Acrylated Epoxidized Soybean Oil and Properties of Resulting Polymers

**DOI:** 10.3390/polym10040439

**Published:** 2018-04-14

**Authors:** Sigita Kasetaite, Silvia De la Flor, Angels Serra, Jolita Ostrauskaite

**Affiliations:** 1 Department of Polymer Chemistry and Technology, Kaunas University of Technology, Radvilenu rd. 19, 50254 Kaunas, Lithuania; sigita.kasetaite@ktu.edu; 2 Department of Mechanical Engineering, Universitat Rovira i Virgili, C/Països Catalans 26, 43007 Tarragona, Spain; silvia.delaflor@urv.cat; 3 Department of Analytical and Organic Chemistry, Universitat Rovira i Virgili, C/Marcel·lí Domingo s/n, Edifici N4, 43007 Tarragona, Spain; angels.serra@urv.cat

**Keywords:** cross-linking, thiol–Michael, soybean oil, bio-based polymer

## Abstract

The effect of the chemical structure and functionality of three structurally different thiols on the cross-linking of acrylated epoxidized soybean oil and on the properties of the resulting polymers was investigated in this study. 1,3-Benzenedithiol, pentaerythritol tetra(3-mercaptopropionate), and an hexathiol synthesized from squalene were used in the cross-linking of acrylated epoxidized soybean oil by thiol–Michael addition reaction. The reactivity of thiols determined from calorimetric curves followed the order: 1,3-benzenedithiol > pentaerythritol tetra(3-mercaptopropionate) > hexathiolated squalene. Thermal and mechanical properties and the swelling in different solvents of the cross-linked polymers were studied. The cross-linked polymer obtained from 1,3-benzenedithiol showed the highest swelling values in chloroform and toluene. The cross-linked polymer with pentaerythritol tetra(3-mercaptopropionate) fragments showed the best mechanical performance (highest mechanical strength and Young’s modulus) and thermal stability. The cross-linked polymers from hexathiolated squalene showed the highest glass transition temperature.

## 1. Introduction

Thiol-click reactions on C–C double bonds are attractive in polymer chemistry due to many advantages, including high reaction rates, quantitative yields, insensitivity to oxygen inhibition, and exhibition of significantly lower levels of shrinkage and stress [1]. Reactions of thiols with alkenes have many applications including high-performance polymers that are important in sensing, optical, and biomedical applications [2,3,4,5,6].

Thiol–ene reactions have been widely used for polymerization of renewable monomers [7,8,9,10], and were referred to the thiol–ene reaction design with UV light initiated coupling reaction involving radical species [11]. As a result of the high reactivity of radical species, some side-reactions occurs. One of these reaction is thiyl–thiyl radical coupling, which leads to disulfide formation; another is head-to-head coupling of the carbon radicals. Both reactions can produce the termination of the thiol–ene cycle. Additionally, the step-mechanism of thiol–ene conjugation is in competition with the chain growth mechanism if a homopolymerizable vinyl monomer is used. In contrast, the thermally activated thiol–Michael addition reaction, which is the conjugate addition of thiols or thiolate anions to electron-deficient C=C bonds, has attracted significant attention of scientists due to its facile and powerful nature and the absence of undesired side-reactions [1] and has been broadly applied in the synthesis of polymeric structures [12,13].

Natural oils are one of the most extensively used renewable feedstocks [14], because of their availability, relatively low cost, chemical functionality, and easy processing [15,16,17,18]. Vegetable oil-based polymers are considered as potentially biocompatible materials [19] because the incorporation of vegetable oil moiety can enhance biodegradation of the materials [20,21]. Soybean oil is the most common vegetable oil source in America, comprising around 57% of all vegetable oil resources [22].

The structure of acrylated epoxidized soybean oil (AESO) has a great interest due to the possibility of polymerizing double bonds from acrylate functional group via free radical reaction using several initiator systems, photoinitiators and UV or visible radiation [23], and high energy radiation such as gamma rays [24]. A less explored possibility [25] is the Michael addition reaction of thiols to these acrylate moieties that proceeds efficiently by the click character of the reaction and the high nucleophilicity of thiols [1,26,27]. This procedure is much easier from the experimental of view because of its thermal conditions used. Moreover, the materials obtained from thiols use to have a high transparency that is needed in some optical applications. One advantage of AESO is its consideration as environment-friendly material, which has been used to obtain polymers and composite products with similar or even better properties than those of petroleum-based polymers [28].

**Scheme 1:**
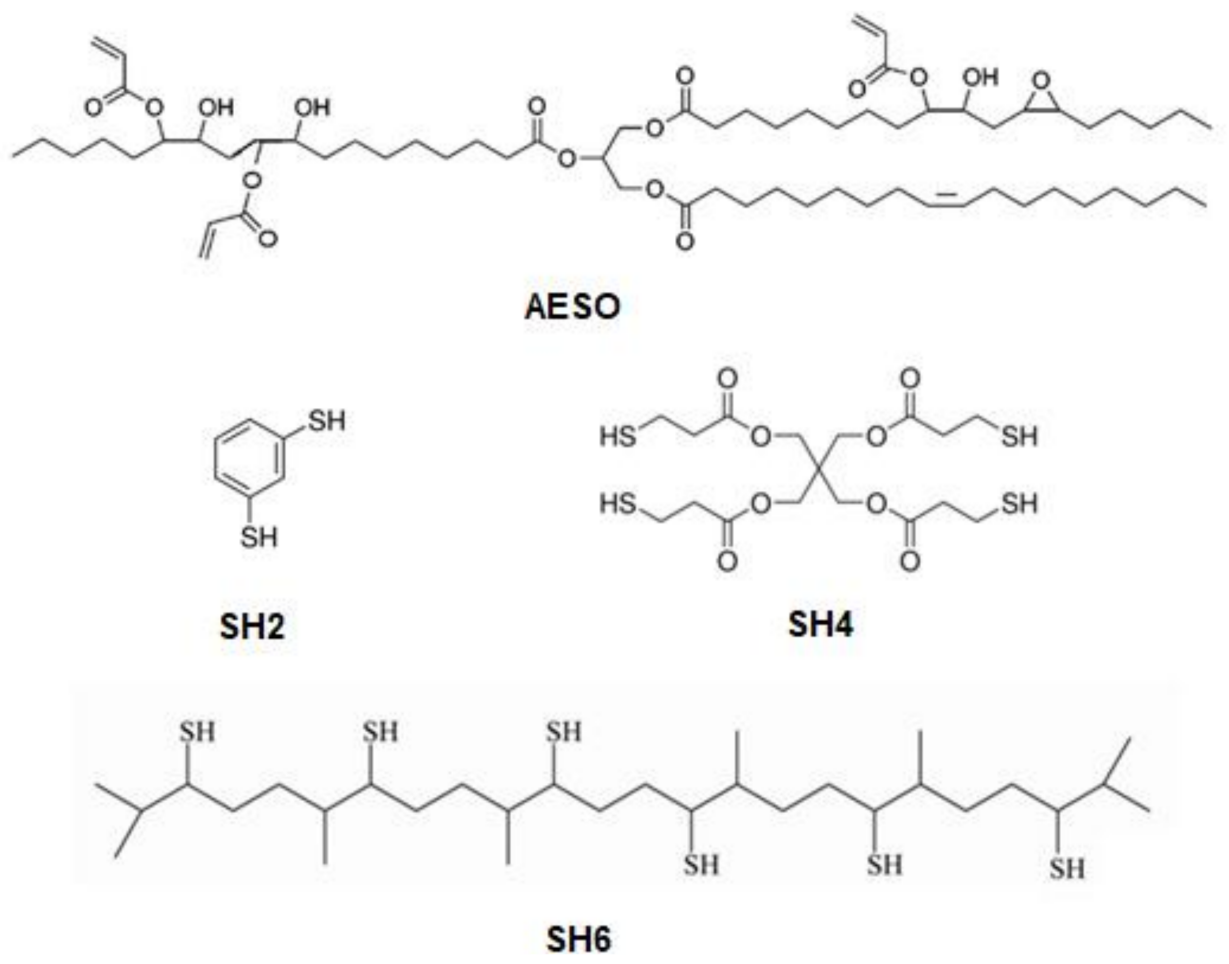
Chemical structures of acrylated epoxidized soybean oil (AESO) and different thiols, 1,3-benzenedithiol (SH2), pentaerythritol tetra(3-mercaptopropionate) (SH4), hexathiol derived from squalene (SH6).

In this study, two commercially available thiols, 1,3-benzenedithiol (SH2) and pentaerythritol tetra(3-mercaptopropionate) (SH4), as well as an hexathiol synthesized from squalene (SH6) were selected for stoichiometric thiol–Michael addition reaction with AESO (Scheme 1). SH4 and SH6 were examined as cross-linking agents in thiol–ene and thiol–epoxy reactions in earlier studies [29,30,31]. In the present study, the effect of the selected thiol structure and functionality on the properties of the resulting polymers has been investigated. To our knowledge, this is the first study on the cross-linked polymers obtained from AESO and different thiols by thiol–Michael addition. The presence of some remaining epoxide groups in the AESO raw material makes the thermal reaction with thiols in the presence of a base more adequate than the previously reported photochemical thiol–ene [23], since epoxide reacts easily with thiols in thermal conditions [26]. The quantitative reaction of thiols with both acrylates and epoxides allows reaching a higher crosslinking density.

The chemical structure of the obtained cross-linked polymers were characterized through infrared (FTIR) spectroscopy. The evolution of the curing process was examined by differential scanning calorimetry (DSC). The thermal characteristics of cross-linked polymers were analyzed by differential scanning calorimetry (DSC), dynamic mechanical thermal analysis (DMTA), and thermogravimetric analysis (TGA). Mechanical characteristics were estimated by tensile tests and hardness measurements. Swelling properties were investigated by measuring the weight of the samples swollen in chloroform and toluene. Although it was reported the photochemical crosslinking by radical species of AESO [23], the materials obtained by our thermal methodology could be much better, since the absence of undesired side-reactions. In addition, thermal processes are quite advantageous in front of photoirradiated since they allow a better control of the reaction and the complete curing of thick samples.

## 2. Materials and Methods

### 2.1. Materials

Acrylated epoxidized soybean oil (AESO, having an average number of acryloyl groups per molecule calculated from ^1^H NMR spectrum as 2.7 and 0.3 of epoxide groups), 1,3-benzenedithiol (SH2), pentaerythritol tetra(3-mercaptopropionate) (SH4), squalene (SQ), 1-methylimidazole (1MI), thioacetic acid (TAA), and 2,2-dimethoxy-2-phenylacetophenone (DMPA) were purchased from Sigma-Aldrich (Darmstadt, Germany). Inorganic salts were recieved from Scharlab (Barcelona, Spain). Methanol and chloroform were purchased from Carlo Erba (Barcelona, Spain). All materials were used without further purification.

### 2.2. Synthesis of Hexathiolated Squalene (SH6)

The product was obtained following a two-step procedure previously reported [32], which includes photochemical thiol–ene coupling reaction of squalene with thioacetic acid and saponification of the resulting thioacetates. The purification of SH6 was carried out by silica gel column chromatography using hexane/ethyl acetate 8/2 mixture as eluent. The yield of the pale yellow viscous liquid was 70%.

^1^H NMR (CDCl_3_, δ in ppm), 2.60 broad (–CH–S–, 6H), 1.10–1.95 unresolved broad signals (–CH_2_–, –CH– and –SH, 32H), and 0.8–1.05 broad (CH_3_–, 24H).

IR (KBr): 2955 (ν CH_2_ aliph.), 2923, (ν C–H aliph.) 2570 (ν S–H) cm^−1^.

### 2.3. Preparation of Cross-Linked Polymers

Several curing formulations were prepared by mixing stoichiometric amounts of AESO with the different thiols, i.e., SH2, SH4, or SH6 (ratio of acrylate/SH groups 1:1). 1 phr (parts of catalyst for hundred parts of total mixture) of 1MI was added as a catalyst. The samples were poured in an 80 mm × 25 mm × 1.5 mm Teflon molds. The curing process was carried out following the schedule: 80, 100, 120, and 150 °C for 1 h, at each temperature to ensure the completion of the thiol–Michael reaction.

### 2.4. Characterizations

#### 2.4.1. Spectroscopic Measurements

A Varian Gemini 400 spectrometer (Palo Alto, CA, USA) was used to register the ^1^H NMR spectra CDCl_3_ was used as the solvent. For internal calibration the solvent signal corresponding to CDCl_3_ was used: δ(^1^H) = 7.26 ppm.

A Perkin-Elmer (Llantrisant, UK) Spectrum BX II FT-IR spectrometer was used to record IR spectra of cross-linked polymers. The spectra were performed in KBr pellets and acquired from 10 scans. The range of wavenumber was (400–4000) cm^−1^.

#### 2.4.2. Soxhlet Extraction

A Soxhlet extractor was used to determine the amount of insoluble polymer fraction. The samples of the cross-linked polymers (0.2 g) were put into a filter package and placed in a Soxhlet apparatus. Extraction was performed with chloroform for 24 h. Insoluble fractions were dried under vacuum to a constant weight. The amount of insoluble fraction was calculated as the difference of the sample weight before and after extraction.

#### 2.4.3. Differential Scanning Calorimetry

A Perkin-Elmer DSC 8500 apparatus was used to determine the evolution and kinetics of the curing process, and the glass transition temperatures (*T*_g_) of the cross-linked polymers. Samples of 10 mg were analysed under non-isothermal conditions in the temperature range from 30 to 250 °C at a heating rate of 10 °C·min^−1^ under nitrogen atmosphere (nitrogen flow rate 100 mL·min^−1^).

The samples were examined at a heating/cooling rate of 10 °C·min^−1^ under nitrogen atmosphere (nitrogen flow rate 50 mL·min^−1^). The *T*_g_ value was taken as the middle point in the heat capacity step of the glass transition.

#### 2.4.4. Thermogravimetric Analysis

Thermal stability of polymers prepared were determined by thermogravimetric analysis (TGA). The measurements were performed on a Perkin-Elmer TGA 4000 apparatus in the temperature range from room temperature to 800 °C at a heating rate of 20 °C·min^−1^ under nitrogen atmosphere (nitrogen flow rate 100 mL·min^−1^).

#### 2.4.5. Dynamic Mechanical Thermal Analysis

Dynamic mechanical thermal analysis (DMTA) was carried out in a bending mode with a TA Instruments (New Castle, DE, USA) DMA Q800 device. Tests were performed with the samples of the following size: 15 mm × 5 mm × 1.5 mm. Single cantilever bending at 1 Hz and amplitude of 10 µm was performed at a heating rate of 3 °C·min^−1^ from −30 to 80 °C. Glass transition temperatures (*T*_g_) were defined as the temperature of the maximum of the peaks of tanδ curves. The rubbery modulus (*E*_r_) values were determined at *T*_g_ + 50 °C from the storage modulus curves. Young‘s moduli were determined at 30 °C under flexural conditions with three point bending clamp, applying a force ramp at constant load rate of 3 N·min^−1^, from 0.001 to 18 N.

#### 2.4.6. Calculation of Cross-Linking Density

The cross-linking density (*ν*_e_) of synthesized polymers was calculated according to The Flory’s rubber elasticity theory [33]

$$\nu_{e}=\frac{E^{\prime }}{3RT_{g}}$$

where *ν*_e_ is the cross-linking density (mol·m^−3^), *E*′ is the apparent rubbery modulus obtained by DMTA from storage modulus curve (Pa), *R* is the universal gas constant (8.314 J·K^−1^·mol^−1^), *T*_g_ is the glass transition temperature of the polymer obtained by DMTA from tanδ curve (K).

#### 2.4.7. Mechanical Testing

Mechanical properties of the cross-linked polymers were estimated by tensile test on a Shimadzu (Duisburg, Germany) AGS-X 10 kN testing machine with a 1 mm·min^−1^ speed according to ASTM 412 standard. Mechanical testing was performed at room temperature on dog bone shaped samples die cut from polymer samples with the dimensions of 80 mm × 25 mm × 1.5 mm. Shore hardness was measured with a durometer type A (Shore A hardness) according to ASTM D2240-05 in samples of, at least, 12 mm in thickness. To obtain the desired thickness the samples were composed of plied pieces. Ten measurements were done in each cross-linked polymer and the average result is presented.

#### 2.4.8. Swelling

The swelling values of cross-linked polymers were estimated by measuring the weight of the samples swollen in chloroform and toluene at room temperature (18 °C). The weighed 20 mm × 20 mm × 1.5 mm samples were placed in a 20 mL vial containing the solvent and stored at room temperature. The samples were taken out every 15 min and weighed after cleaning their surfaces. The arithmetic average of three swollen samples of each polymer was calculated.

## 3. Results and Discussion

### 3.1. Preparation, Characterization of Cross-Linked Polymers, and Study of Curing Process

The chemical reaction involved in the curing process is the well know thiol–Michael addition. This reaction starts by the attack of a nucleophile, in this case thiolate anion, to the double bond of the acrylate group on the conjugated position. The formation of the thiolate requires the use of a base, in general a tertiary amine as the catalyst. In the present study 1MI was selected, in a proportion of 1 phr, after preliminary calorimetric tests. 1MI is also a good catalyst for thiol–epoxy click reaction, which is overlapped with thiol–Michael addition.

The cross-linked polymers were obtained by thermal polymerization of AESO with stoichiometric amounts of different thiols using 1MI as a catalyst. The resulted materials were transparent, soft, and had a yellowish color.

The cured materials were studied by FTIR, which shown the typical absorptions collected below and the complete disappearance of the acrylate at 1637 cm^−1^ and the weak –S–H (at 2633–2565) absorptions:

IR (KBr) of cross-linked polymer AESO/SH2: 3339 (ν O–H), 2923 (ν CH_2_ aliph.), 1734 (ν C=O), 1166 (ν C–O–C) cm^−1^.

IR (KBr) of cross-linked polymer AESO/SH4: 3568 (ν O–H), 2933 (ν CH_2_ aliph.), 1735 (ν C=O), 1157 (ν C–O–C) cm^−1^.

IR (KBr) of cross-linked polymer AESO/SH6: 3422 (ν O–H), 2853 (ν CH_2_ aliph.), 1733 (ν C=O), 1171 (ν C–O–C) cm^−1^.

Due to the low content of epoxides in AESO and their weak typical absorption bands in the FTIR spectrum, together with the overlapping of the bands of ether moieties in the AESO spectrum, no changes related to the thiol–epoxy reaction could be observed.

**Figure 1:**
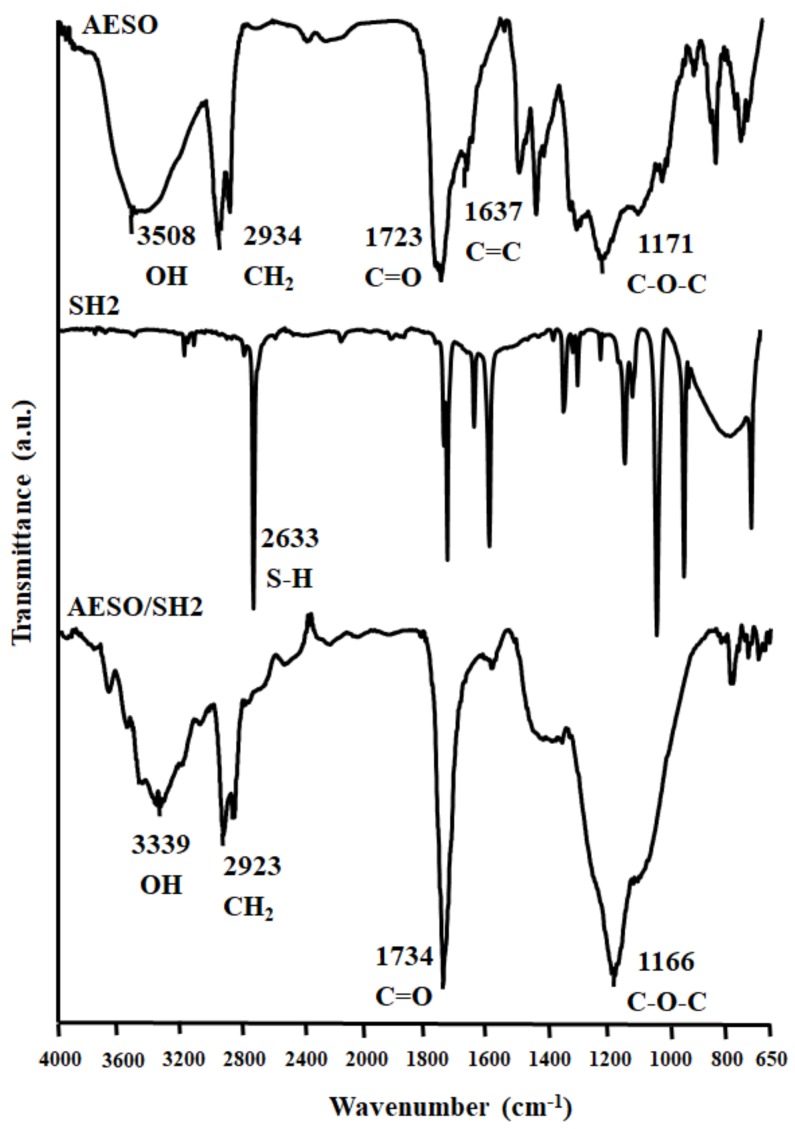
IR spectra of acrylated epoxidized soybean oil (AESO), 1,3-benzenedithiol (SH2), and cross-linked polymer AESO/SH2.

As an example, FTIR spectra of AESO, SH2, and cross-linked polymer AESO/SH2 are presented in Figure 1.

**Figure 2:**
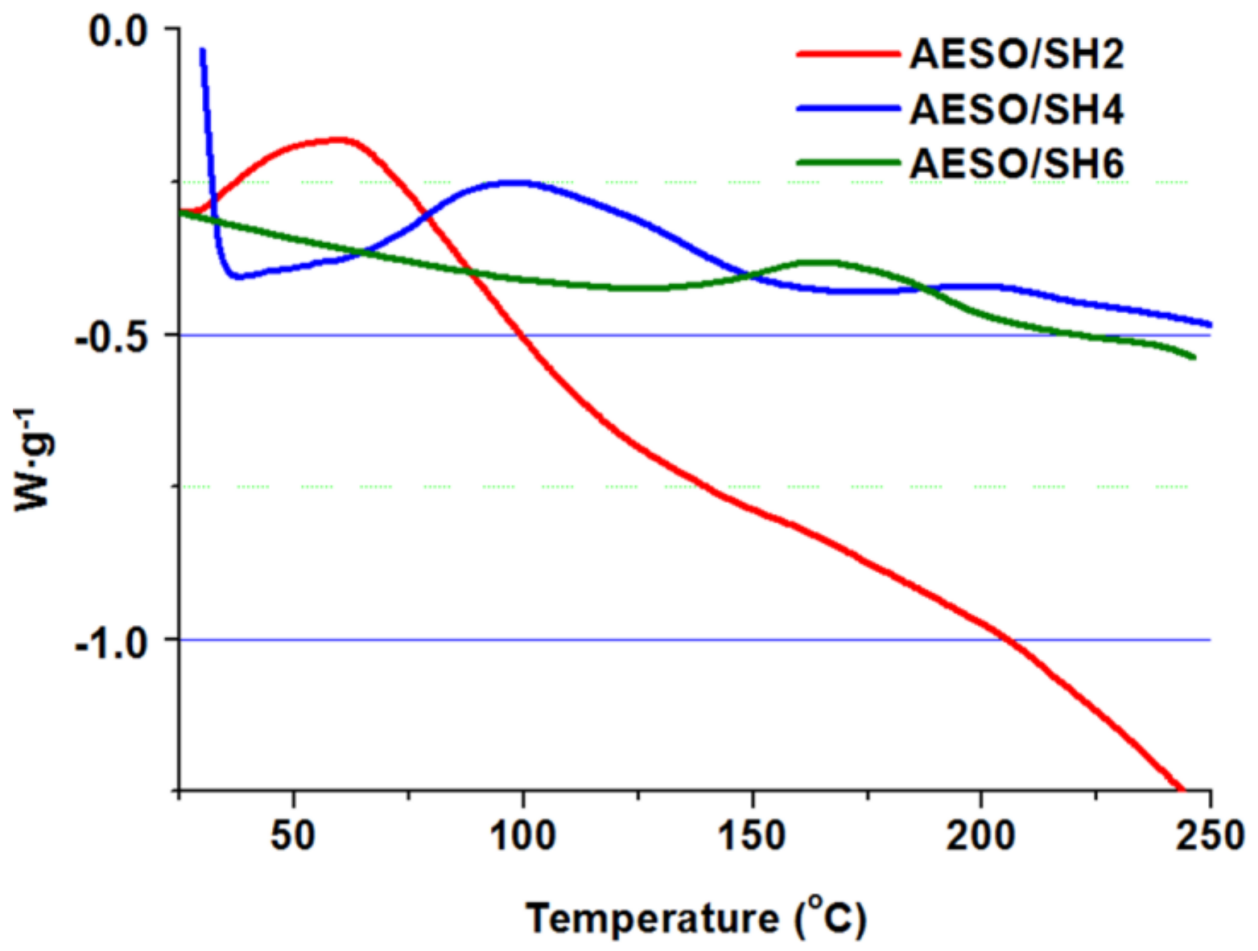
Calorimetric curves of the curing of AESO with different thiols with 1 phr of 1MI recorded at 10 °C·min^−1^.

The evolution of the curing process was examined by calorimetric studies. As an example, calorimetric curves of different compositions with 1 phr of 1MI are presented in Figure 2. The cross-linking of AESO with SH2 starts at room temperature and the curve is broad. This indicates that from the very beginning thiolate groups are formed due, to the highest acidity of the aromatic thiols in reference to the other aliphatic thiols. These thiolates have a high nucleophilicity in the conjugate addition. The cross-linking of AESO with SH4 starts at a higher temperature than the reaction with SH2 and the shape of the curve is narrower and higher, which indicates that once activated, the system becomes more reactive than the other ones. The reaction of AESO with SH6 starts at 130 °C and finishes at high temperature which indicates that the reactivity is rather low. This can be explained by its structure with a high functionality and secondary thiol moieties, and it agrees with other studies performed previously [31,34]. Values of enthalpy and temperatures of the calorimetric peak maximum of the curing of AESO compositions with different thiols and 1MI as the catalyst are collected in Table 1. The biggest amount of heat (142.7 J·g^−1^) was released during the curing of AESO with SH2. While the lowest amount of heat (20.1 J·g^−1^) was released during the curing of AESO with SH6. The curing enthalpies given are only approximate, due to the shape of the curves. The temperature of the maximum of the calorimetric peak for the curing of AESO with SH6 reached 170 °C and for the curing of AESO with SH2 reached 64 °C. According to that, the determined reactivity of the used thiols in the cross-linking of AESO was as follows: SH2 > SH4 > SH6.

The materials obtained from AESO and all the thiols selected were insoluble in all the solvents tested, confirming their crosslinked character. The yield of insoluble fraction of the polymers was determined by Soxhlet extraction. The results obtained are collected in Table 1. Insoluble fractions were higher than 85% in all cases and the materials AESO/SH2 and AESO/SH6 led to a lower value than those of the polymer AESO/SH4. The determined values are related with the structural characteristics of thiols. It was expected that the polymer AESO/SH6 could show the highest value of yield of insoluble fraction due to the highest number of thiol groups in SH6 that should lead to a more compact structure, with less soluble fraction. The value obtained seems to indicate an incomplete curing, that will be confirmed by DMTA in the next section. SH6 has a theoretical functionality of 6, according to its 6 thiol groups, but a compact structure with a short distance among thiol groups and a quite hindered position of these groups in the whole molecular structure. These characteristics can lead to serious topological restrictions leading to an incomplete reaction and therefore to an undesired imbalance between thiol and acryloyl groups. Incomplete curing in case of using SH6 as thiol has been reported previously [30,31,34]. In case of AESO/SH2, a low yield of insoluble fraction was also measured. This is probably due to the lower functionality of SH2 that leads to a reduced degree of cross-linking. The use of SH4 led to the higher yield of insoluble fraction of the polymer AESO/SH4 than the use of other thiols due to the more flexible structure and longer distance between thiol groups in SH4 molecule that allows a quantitative reaction of thiol groups with AESO. As we can see in the table, there is not much difference between the *T*_g_s determined for the different materials. These *T*_g_s are rather low according to their elastomeric character at room temperature.

### 3.2. Thermomechanical Properties

Thermal characteristics of the polymers are of the main importance in their applications. Thus, thermomechanical and thermal stability studies are usually performed when characterized. Thermal and dynamic mechanical analysis data of cross-linked polymers are collected in Table 2. Although we expected a higher *T*_g_ for SH6 derived materials, because of the high functionality, the result seems to indicate that not all the SH groups have reacted and the effective functionality is lower than 6, as it was previously noticed in the curing of cycloaliphatic resins [30]. The presence of an aromatic structure in SH2 would led to a high *T*_g_ value but this effect is compensated by the low functionality of this monomer. On comparing the *T*_g_ values for the materials AESO/SH4 obtained by photochemical thiol–ene [23] and thermal thiol–Michael reactions, we can state that a slightly higher value was reached thermally (−1.3 °C in front of 3 °C).

**Figure 3:**
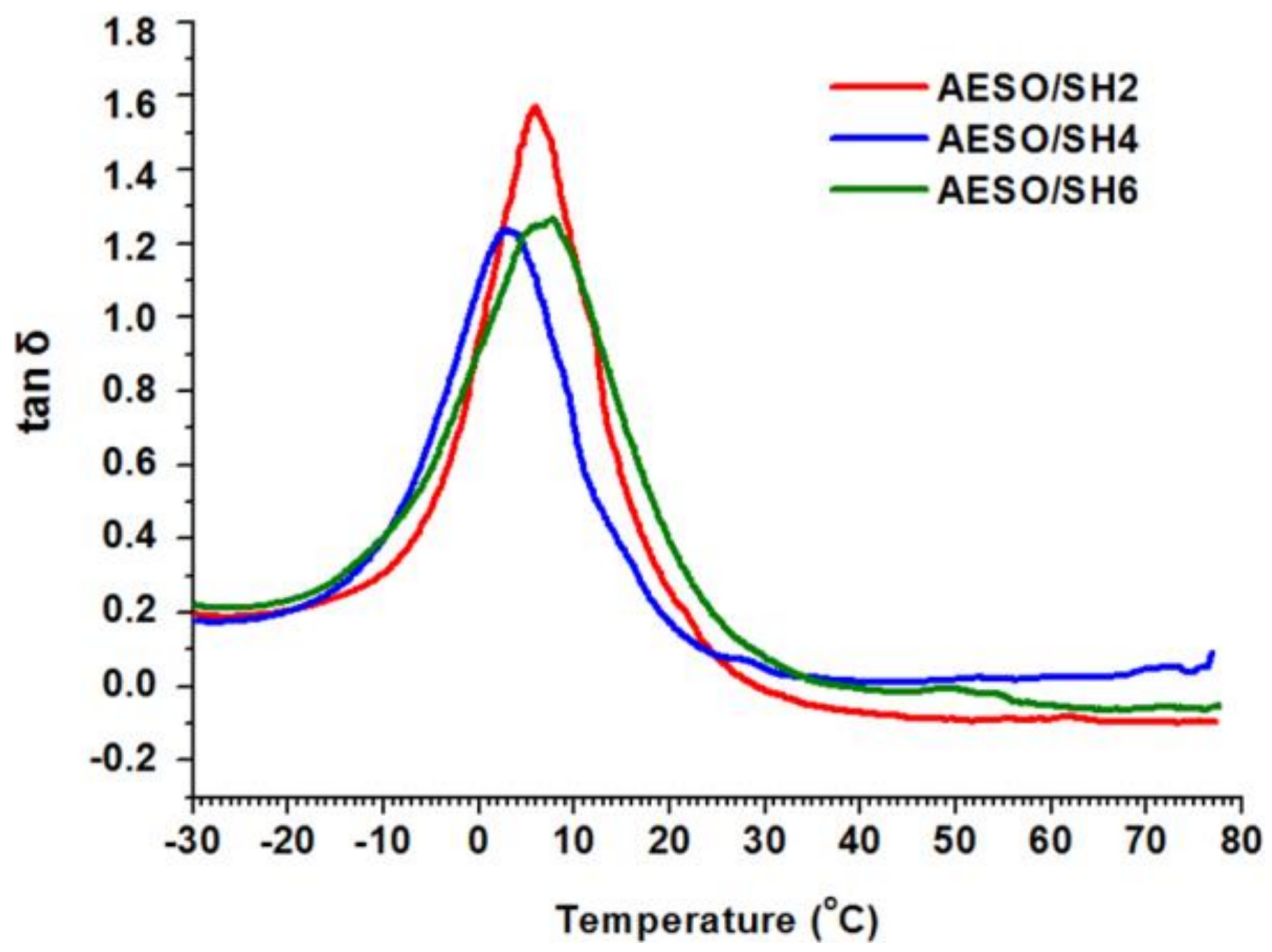
The tanδ versus temperature curves of cross-linked polymers prepared from different thiols.

The tanδ versus temperature curves of cross-linked polymers with different thiols are shown in Figure 3. The tanδ curves of the cross-linked polymers have a similar narrow shape, which indicates a high homogeneity of the network structure. The curve of the cross-linked polymer AESO/SH2 has the highest intensity due to the less crosslinked structure and the lower functionality of SH2 that affect damping characteristics. The tanδ curves of polymers AESO/SH4 and AESO/SH6 are analogous in shape and range which seems to confirm that the effective functionalities of both thiols have similar values.

**Figure 4:**
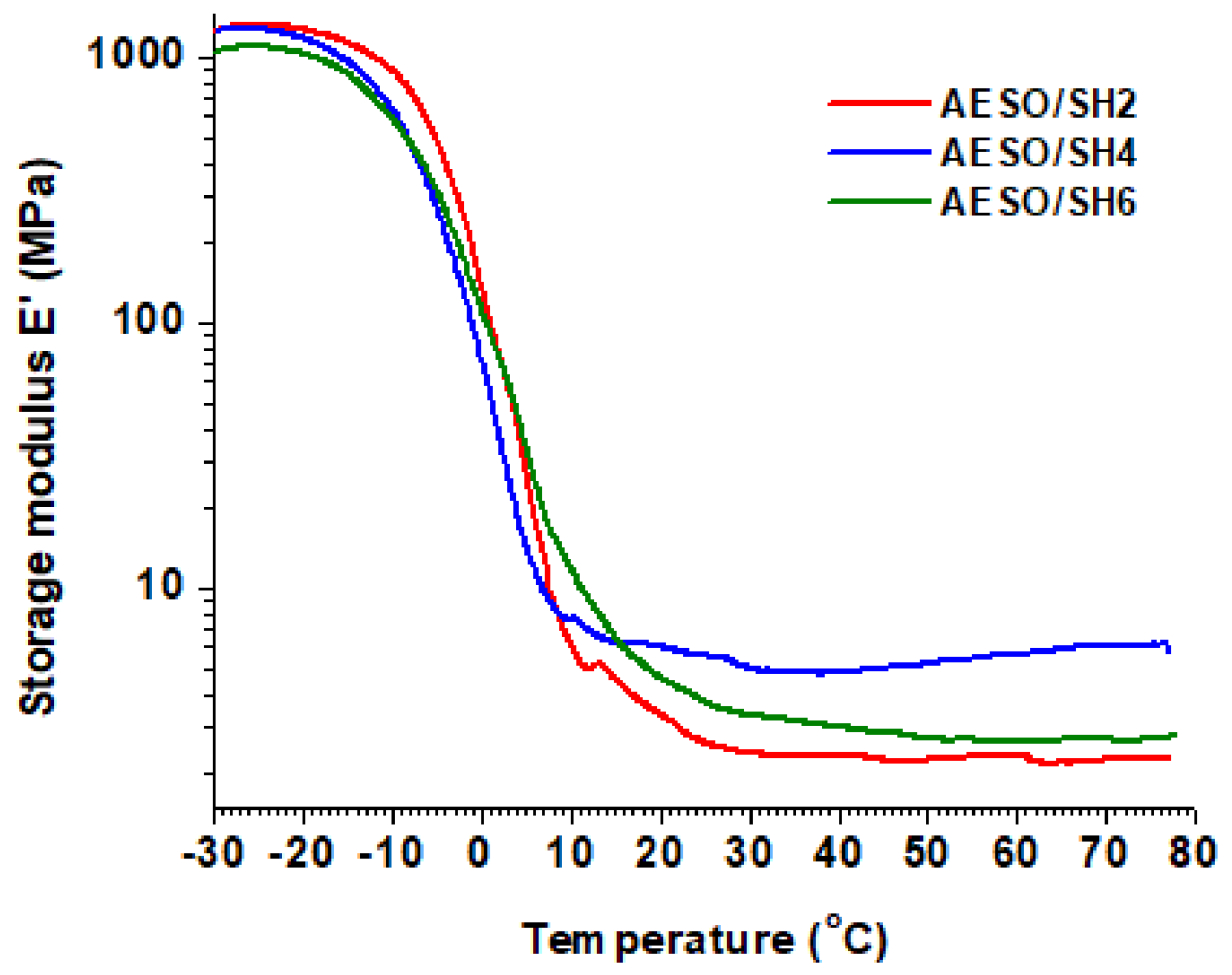
The storage modulus *E*′ versus temperature curves of the cross-linked polymers prepared.

Figure 4 shows the storage modulus (*E*′) curves versus temperature of the cross-linked polymers. The storage moduli in the rubbery state are collected in Table 2. Applying the Flory‘s equation (see Materials and Methods part), we calculated the cross-linking densities for each material. From these values the highest rubbery modulus and the highest degree of cross-linking correspond to AESO/SH4 material. This is in accordance with the highest amount of insoluble fraction given in the previous section. From these results we can conclude that the most effective thiol to get the highest cross-linked polymer is SH4. The material obtained from SH2 has the lowest rubbery modulus according to its lowest functionality and again SH6 seems to have an intermediate effective functionality between functionalities of SH2 and SH4, lower than the theoretical one. The Young’s modulus values determined by DMTA in flexural mode show a similar trend, although with a different magnitude, due to the differences in temperature and in the measurement mode (static in front of dynamic). The cross-linked polymer containing SH2 has the lowest Young’s modulus value at 30 °C (92 kPa) and the cross-linked polymers containing SH4 and SH6 have a quite similar Young’s modulus values (325 and 294 kPa, respectively).

The values of the crosslinking density obtained from the relaxed modulus and the Flory’s rubber elasticity theory reveal a higher crosslinking density for the material obtained from SH4, which is comparable or even slightly higher than the crosslinking density obtained by photochemical thiol–ene curing (794 mol·mm^−3^) [23].

**Figure 5:**
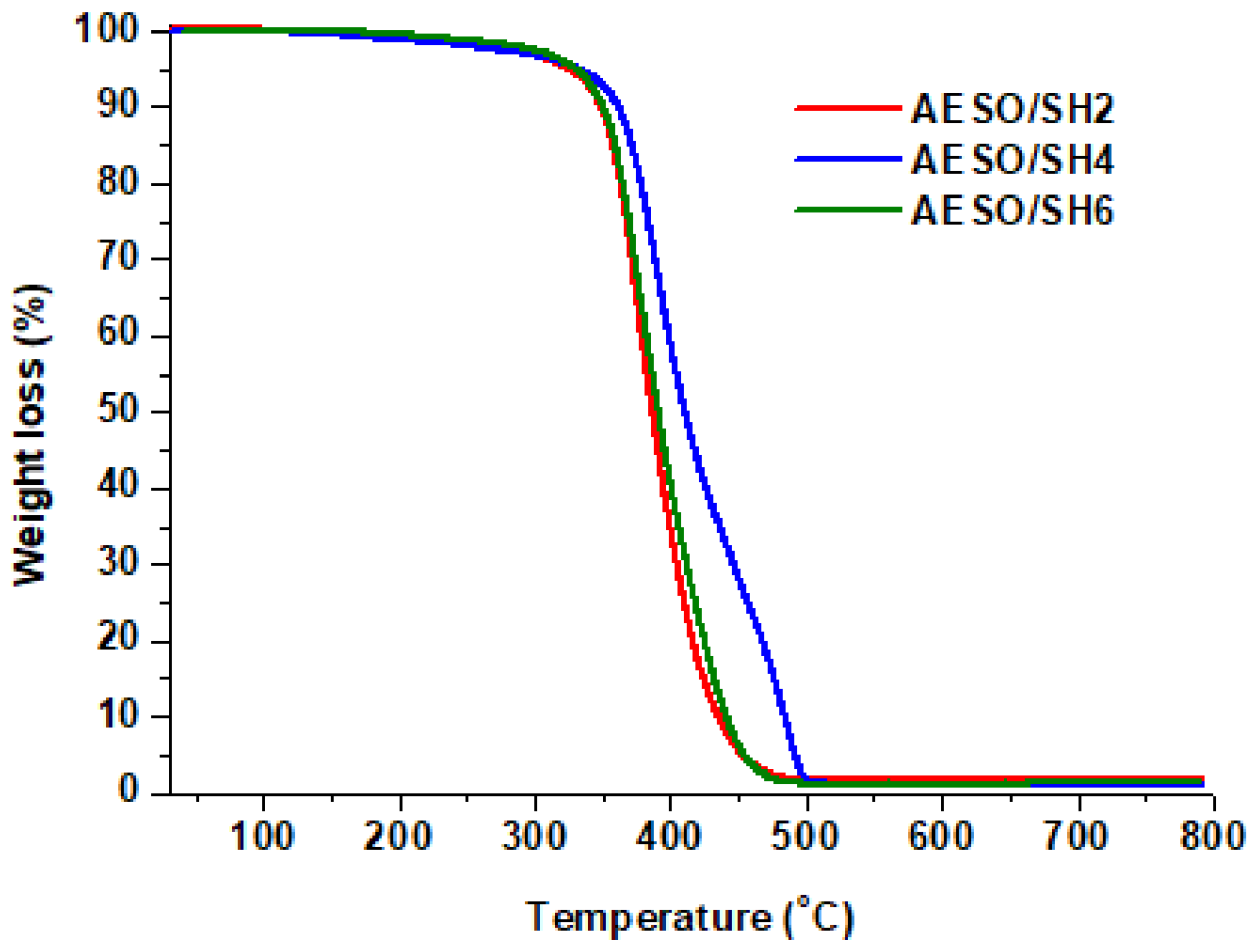
Thermogravimetric curves of polymers cross-linked with different thiols.

TGA was used to evaluate the thermal stability of cross-linked polymers. Thermal decomposition of all polymers occurs in a very sharp manner and proceeded in an only step for materials obtained from SH2 and SH6 (Figure 5). However, the shape of the AESO/SH4 curve shows the presence of a slight shoulder at higher temperatures. The presence of ester groups in SH4 moieties could be the responsible of this different behavior. The highest temperature of 10% weight loss (*T*_dec-10%_) was determined for the polymer AESO/SH4 (361 °C) (see Table 2), which could be correlated to the highest cross-linking density. The polymers AESO/SH2 and AESO/SH6 show similar *T*_dec-10%_ values (349 and 350 °C, respectively). All the residues at 800 °C are very low due to the aliphatic character of the network, although the highest residue corresponds to AESO/SH2, which contains aromatic fragments.

### 3.3. Mechanical Properties

**Figure 6:**
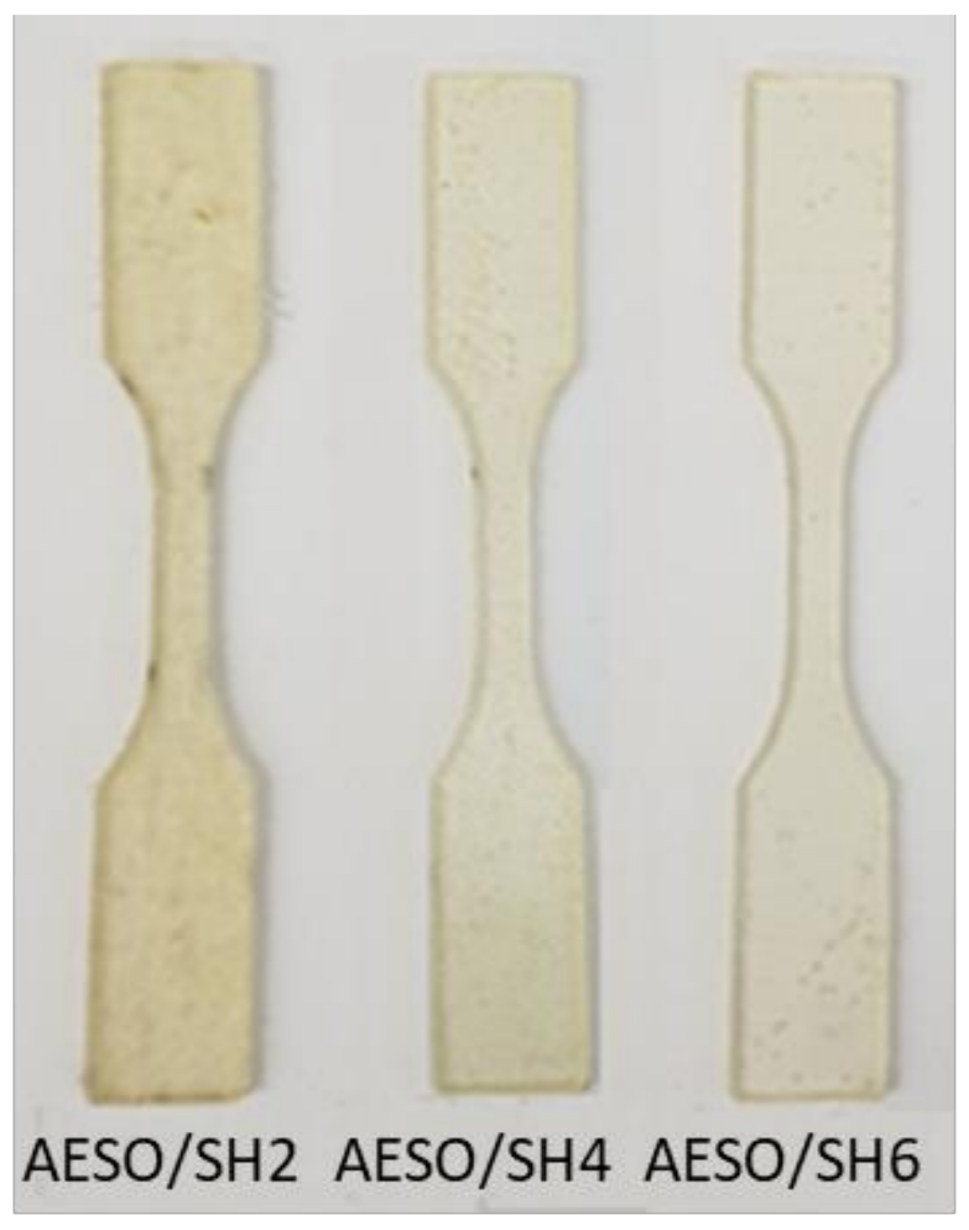
Images of the dog-bone shaped samples die cut from polymer samples.

The mechanical characterization of the prepared materials is indispensable in designing their future applications. The images of the dog-bone shaped samples prepared for the tensile test are shown in Figure 6.

Table 3 shows the mechanical data obtained from the stress-strain curves and hardness measurements for all the samples. From the inspection of the values we can see two different behaviors: one, more flexible, with higher ductility, lower rigidity and lower hardness corresponding to the material obtained from SH2, which is the less crosslinked, and the other, that includes the materials with SH4 and SH6 moieties, with a higher mechanical performance. From this group, the elastomer AESO/SH4 presents the highest tensile strength (0.54 MPa) and tensile modulus (3.8 MPa) because of the apparently highest functionality in comparison to SH6 [31]. All these results are in accordance with those obtained from DMTA tests and presented in Table 2. Results for AESO/S4 are also very similar with those reported for Wang et al. [23].

### 3.4. Swelling Properties

**Figure 7:**
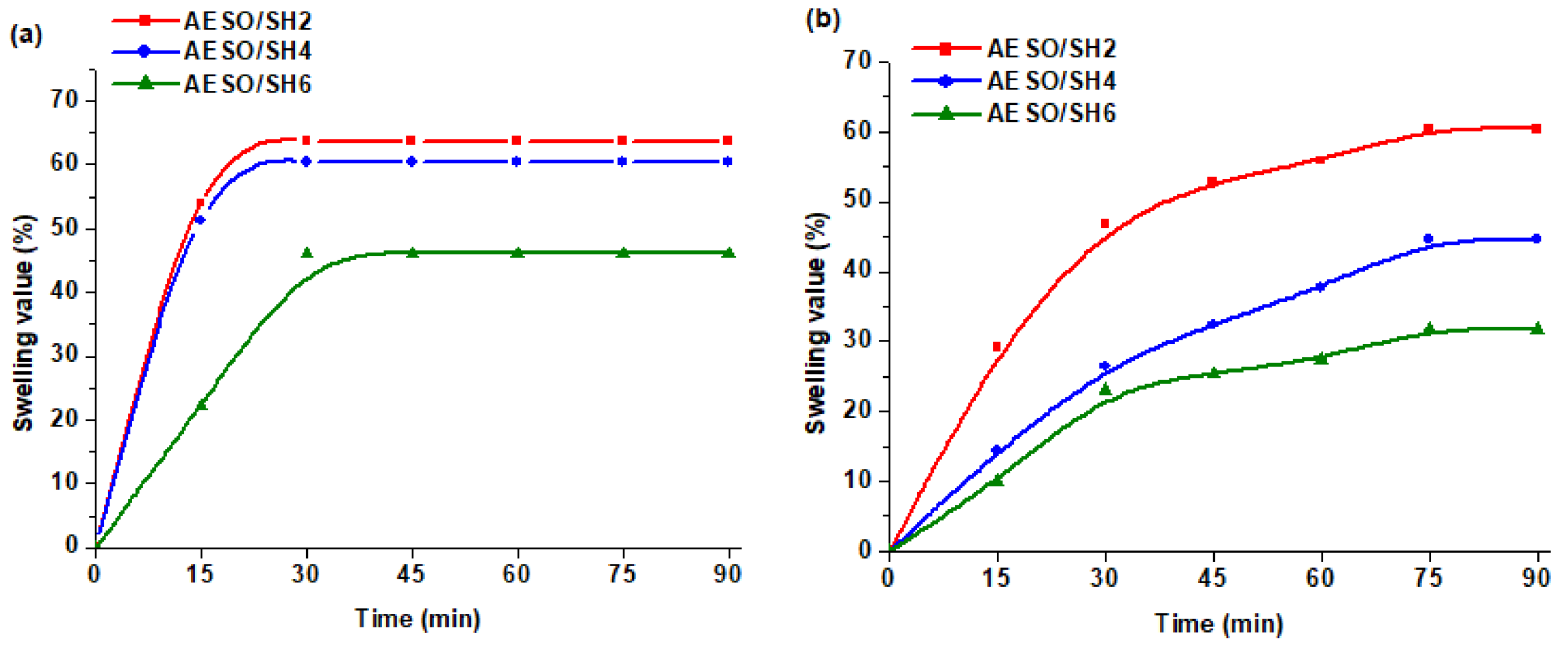
Swelling values: (**a**) in chloroform; (**b**) in toluene at 18 °C versus times curves of cross-linked polymers.

Crosslinking densities are related to swelling capacities in networked polymers and therefore swelling tests are of great importance to characterize the network structure. Swelling value versus time curves obtained from swelling test are presented in Figure 7. The cross-linked polymers swelled in choroform and toluene in the following order: AESO/SH2 > AESO/SH4 > AESO/SH6. The highest swelling values in chloroform and toluene (64% and 61%, respectively) were obtained for polymer AESO/SH2 showing that longer chains between the cross-linking points were formed in this polymer as the result of the lower functionality of SH2. This observation correlates with the calculated cross-linking density of polymer AESO/SH2 which was the lowest between all polymers investigated in this study. The lowest swelling values in chloroform and toluene (46% and 32%, respectively) were obtained for polymer AESO/SH6, whereas the material obtained from SH4 has an intermediate swelling value. This result seems to be contradictory to the crosslinking density values calculated, higher for SH4 than for SH6. However, some other factors affect the swelling properties, such as the polarity of the structure that affects the polymer-solvent interactions. SH4 moieties has a higher polarity than SH6, because of the presence of ester groups and therefore the material obtained from SH4 has a better interaction with the solvent, much better with chloroform, which has a higher polarity.

## 4. Conclusions

Novel bio-based cross-linked polymers were synthesized from acrylated epoxidized soybean oil and thiols with different functionality by a thermal click thiol–Michael addition reaction. The determined reactivity of thiols in the cross-linking of the acrylated oil was as follows: 1,3-benzenedithiol > pentaerythritol tetra(3-mercaptopropionate) > hexathiolated squalene. The use of 1,3-benzenedithiol with the functionality of 2 led to the cross-linked polymer with the lowest values of insoluble fraction and cross-linking density, and the highest swelling values in chloroform and toluene due to the presence of fewer cross-linking points in the network. The use of pentaerythritol tetra(3-mercaptopropionate) with the functionality of 4 led to the cross-linked polymer with the highest insoluble fraction, cross-linking density, thermal stability and the best mechanical performance. Hexathiolated squalene shows an effective functionality lower than 6, probably due to the steric hindrance and lower reactivity to the attack of the thiol groups to the acrylate moieties, that leads to intermediate thermal and mechanical properties, but similar to those obtained from SH4 thiol. However, this derivative is obtained from a renewable compound and therefore the materials obtained can be considered as fully renewable.

## Figures and Tables

**Table 1:** Reaction enthalpies, temperatures of the curing exotherm maximum, and glass transition temperatures for the different formulations yields of insoluble fraction of cross-linked polymers.

Polymer	Δ*H* ^1^ (J·g^−1^)	*T*_max_^2^ (°C)	*T*_g_^3^ (°C)	Yield of insoluble fraction ^4^ (%)
AESO/SH2	142.7	64	−12	86
AESO/SH4	63.3	101	−12	90
AESO/SH6	20.1	170	−11	87

^1^ Enthalpy released per gram on heating the initial mixture; ^2^ Temperature of the curing exotherm maximum; ^3^ Glass transition temperature estimated by DSC; ^4^ After Soxhlet extraction with chloroform for 24 h.

**Table 2:** Thermal and dynamic mechanical analysis data of cross-linked polymers.

Polymer	DMA				TGA	
	*T*_tanδ_^1^ (°C)	*E*_r_^2^ (MPa)	*ν*_e_^3^ (mol·m^−3^)	*E* ^4^ (kPa)	*T*_dec-10%_^5^ (°C)	Res. ^6^ (%)	
AESO/SH2	6	2.3	325	92	349	2	
AESO/SH4	3	5.5	844	325	361	1	
AESO/SH6	8	3.3	400	294	350	1	

^1^ Temperature of the maximum of tan δ estimated by DMTA; ^2^ Storage modulus in the rubbery state determined at *T*_g_ +50 °C; ^3^ Cross-linking density calculated according to the Flory’s rubber elasticity theory; ^4^ Young‘s modulus at 30 °C under flexural conditions; ^5^ Temperature at the weight loss of 10% obtained from TGA curves; ^6^ Residue yield after thermal degradation in N_2_ atmosphere.

**Table 3:** Mechanical characteristics of cross-linked polymers.

Polymer	Strain at break (%)	Stress at break (MPa)	Tensile modulus (MPa)	Shore A hardness
AESO/SH2	19.4	0.26	1.6	39
AESO/SH4	15.6	0.54	3.8	62
AESO/SH6	14.8	0.44	3.3	62
